# WORKPLACE EMOTIONAL EXPERIENCES, TIME SAVORING, AND HELPING BEHAVIORS IN AN AGE-DIVERSE SAMPLE OF ADULTS

**DOI:** 10.1093/geroni/igad104.1818

**Published:** 2023-12-21

**Authors:** Claire Growney, Li Chu, Laura Carstensen

**Affiliations:** Stanford University, Stanford, California, United States; Stanford University, Stanford, California, United States; Stanford, Stanford, California, United States

## Abstract

Socioemotional selectivity theory suggests that as future time perspective grows limited with age, older adults place greater priority on emotionally meaningful experiences and social relationships. Older adults are also theorized to savor time spent in emotionally meaningful activities and social interactions. A preliminary study found that age was associated with higher experience of positive emotion at work. In addition, we found that age was positively associated with trait-level reports of time-savoring, which in turn predicted higher engagement in helping behaviors. We will present findings from a study utilizing the day reconstruction method (DRM) that examines associations between time-savoring at work and engagement in positive workplace behaviors in full-time workers. For one full workweek, participants (N = 200, aged 25–80) complete a survey at the end of each day asking about their workdays parsed into three segments. They report on their social and emotional experiences, the extent to which they were savoring their time engaged in different workplace activities, and their helping (e.g., providing advice to a co-worker) and learning (e.g., developing new ideas or solutions to problems) behaviors during each segment of their workday. Findings have implications for workplace productivity in increasingly age-diverse workplaces. Time-savoring at work may be conducive to intergenerational transfers of knowledge.

